# Study on MEV90 of 0.5% ropivacaine for US-guided caudal epidural block in anorectal surgery

**DOI:** 10.3389/fmed.2022.1077478

**Published:** 2023-01-19

**Authors:** Pei Zhang, Hong Chang, Taoran Yang, Yalong Fu, Xuemei He, Jun Li, Mingan Yang, Rurong Wang, Xuehan Li

**Affiliations:** ^1^Department of Anesthesiology, Laboratory of Anesthesia and Intensive Care Medicine, West China Hospital of Sichuan University, Chengdu, Sichuan, China; ^2^Department of Anesthesiology, West China Hospital, Sichuan University, Chengdu, China; ^3^West China School of Nursing, Sichuan University, Chengdu, China; ^4^Chengdu Shang Jin Nan Fu Hospital, Chengdu, China; ^5^Department of Anus Intestine, Karamay People's Hospital, Xinjiang, China; ^6^Division of Biostatistics and Epidemiology, School of Public Health, San Diego State University, San Diego, CA, United States

**Keywords:** anorectal surgery, caudal block, ropivacaine, minimum effective volume, ultrasound

## Abstract

**Background:**

Choosing the appropriate concentration and volume of anesthetics is critical for a successful nerve block. The current study aimed to determine the minimum effective volume (MEV) of 0.5% ropivacaine for US-guided CEB in 90% of patients (MEV90) undergoing anorectal surgery. The aims were to reduce the occurrence of complications associated with a sacral blockade in anorectal surgery, broaden the indications for surgical procedures and treatment, and improve patient satisfaction. This study presents the groundwork for the development of individualized anesthetic programs. We believe that the study would serve as a reference for the use of caudal epidural block (CEB) in lower abdominal surgery for intraoperative and postoperative analgesia.

**Methods:**

This study used a biased coin design (BCD) up-and-down method (UDM). We divided the participants into two groups based on gender, and each group independently performed the biased coin design up-and-down method. We used 0.5% ropivacaine for the first patient in each group; however, the volume was 10 ml for men and 8 ml for women. Therefore, the dose of anesthetics given to each patient was determined by the response of the previous patient. If the block of the previous patient failed, the volume was increased by 2 ml in the following patient. Otherwise, the next subject had an 11% chance of receiving a volume of 2 ml less or an 89% chance of receiving no volume change. We defined a successful block as painless surgery with anal sphincter relaxation 15 min after the drug injection. Enrollment was completed after 45 successful caudal blocks for each group.

**Results:**

Caudal epidural block was successfully performed on 50 men and 49 women. The MEV90 of ropivacaine for CEB was calculated to be 12.88 ml (95% CI: 10.8–14 ml) for men and 10.73 ml (95% CI: 9.67–12 ml) for women. Men had a MEV99 of 13.88 ml (95% CI: 12.97–14 ml), and women had a MEV99 of 11.87 ml (95% CI: 11.72–12 ml).

**Conclusion:**

With operability and general applicability, it is possible to increase the success rate of CEB for anorectal surgery to 99% as well as decrease the incidence of anesthesia-related complications. CEB can meet the needs of patients for rapid postoperative rehabilitation, improve patient satisfaction, and lay a solid foundation for postoperative analgesia.

## 1. Introduction

With the expanding indications for anorectal surgery and the growing need for comfortable medical care, the need for effective anesthesia and postoperative analgesia is growing. With the use of ultrasound in the CEB (1, 2), precision anesthesia has become the mainstream direction of regional anesthesia. Therefore, the minimum effective volume and concentration of CEB (2–4) must be well-defined, particularly to reduce the incidence of complications directly related to local anesthetics.

Caudal epidural block (CEB) provides reliable anesthesia for the lumbosacral nerve roots and has recently become popular in adult anorectal surgery (3). Ropivacaine, one of the most commonly used local anesthetics in the CEB, has been shown to separate sensory and motor blocks better than bupivacaine or lidocaine (5, 6). The MEC50 of ropivacaine in CEB was identified to be 0.296% in men and 0.389% in women using Dixon's up-and-down method (UDM), which is a common method for determining the minimum effective volume/concentration in 50% of patients (MEV50). Most studies also reported that 0.5% ropivacaine was the most commonly used concentration in caudal anesthesia, with a volume range of 10–25 ml (7, 8); however, the CEB of this volume of ropivacaine for anorectal surgery is insufficiently precise, and its clinical application is limited (9, 10). Complications with high-volume epidural injections have been reported, including the excessive plane of the block, dyskinesia of both lower limbs, and urinary retention, which delayed the rapid postoperative recovery of patients and reduced patient satisfaction. Low-volume epidural injections result in block failure, necessitating a change in anesthetic modality (11).

Determining the MEV of ropivacaine to ensure the block effect and avoid side effects caused by an overdose of local anesthetics or to avoid anesthesia failure due to insufficient local anesthetic volume is of great clinical importance. However, the 90% MEV (MEV90) of ropivacaine for caudal block in anorectal surgery is unknown in adults. MEV90 is a more precise reference for the application of sacral block to local anesthesia in anorectal surgery than MEV50 (3, 12). Therefore, the current study was designed to investigate the MEV90 of 0.5% ropivacaine for US-guided CEB in adults undergoing anorectal surgery using a biased coin design UDM. Furthermore, as an important tool for CEB, ultrasound can not only perform preoperative sacral canal evaluation but also guide the puncture needle through the sacral ligaments in real time into the sacral canal cavity, ensuring that all anesthetics are injected into the sacral canal (2, 4). Men require less ropivacaine for caudal anesthesia than women, according to previous research (9). Therefore, the trial for the current study was conducted separately in men and women. The current study aimed to determine the MEV90 of ropivacaine in men and women undergoing anorectal surgery using US-guided CEB (9, 11), improving CEB success rates while decreasing the incidence of associated complications, thus providing a better demonstrative effect for all peripheral regional blocks.

## 2. Materials and methods

The protocol of this study was approved by the Clinical Trial Ethics Committee of Chengdu Shangjin Nanfu Hospital (No. 2019042506) and is now registered in the Chinese Clinical Trial Center (No. ChiCTR 1900024315). Before enrollment, all patients signed informed consent forms. After the clinical trial was registered, patient enrollment began in October 2019. The study adhered to the principles enshrined in the Helsinki Declaration, and this manuscript follows the relevant CONSORT guidelines.

### 2.1. Patient enrollment

This study included patients who underwent anorectal surgery (hemorrhoidectomy, anal fistula resection, and perianal abscess) at Chengdu Shangjin Nanfu Hospital between 1 October 2019 and 6 January 2020. Patients with a BMI of <30 kg/m^2^ and an American Society of Anesthesiologists (ASA) Grade I—II were eligible. Sonographic evidence of sacral stenosis or occlusion (the anteroposterior diameter of the sacral hiatus was <1.6 mm), participation in other clinical studies within 3 months, allergy or contraindication to local anesthetic amides, coagulopathy, severe hepatic or kidney dysfunction, history of infection or surgery in the sacrococcygeal region, and inability to sign consent forms were the main exclusion criteria.

Preoperatively, all patients who took part in the study signed written informed consent. In our hospital, the anesthesia options for anorectal surgery included general anesthesia and CEB. Patients were informed before surgery about the various types of anesthesia that could be used during anorectal surgery. CEB was preferred if they agreed to participate in the study. Patients were also informed that, if the CEB fails, they may be subjected to general anesthesia.

#### 2.1.1. US-guided caudal epidural block

As CEB took a long time to prepare, patients were sent to a prep room 30 min before surgery equipped with monitoring equipment, rescue drugs, and a local anesthetic system toxicity (LAST) kit with the consent of the patient and surgeon. All procedures were carried out with real-time ultrasound guidance (Mindray 7, Shenzhen, China).

The patient was admitted to the ward with an 18G intravenous line, and 500 lactated Ringer's solution was administered intravenously. Following the arrival of the patient in the prep room, ECG, non-invasive pulse blood, and pulse oximetry were routinely monitored, and oxygen was administered *via* a nasal catheter at a rate of 4 L/min. Following preparation, the patients were placed in a left lateral position, and an experienced anesthesiologist performed CEB under real-time ultrasound guidance (3, 13).

**Figure 1:**
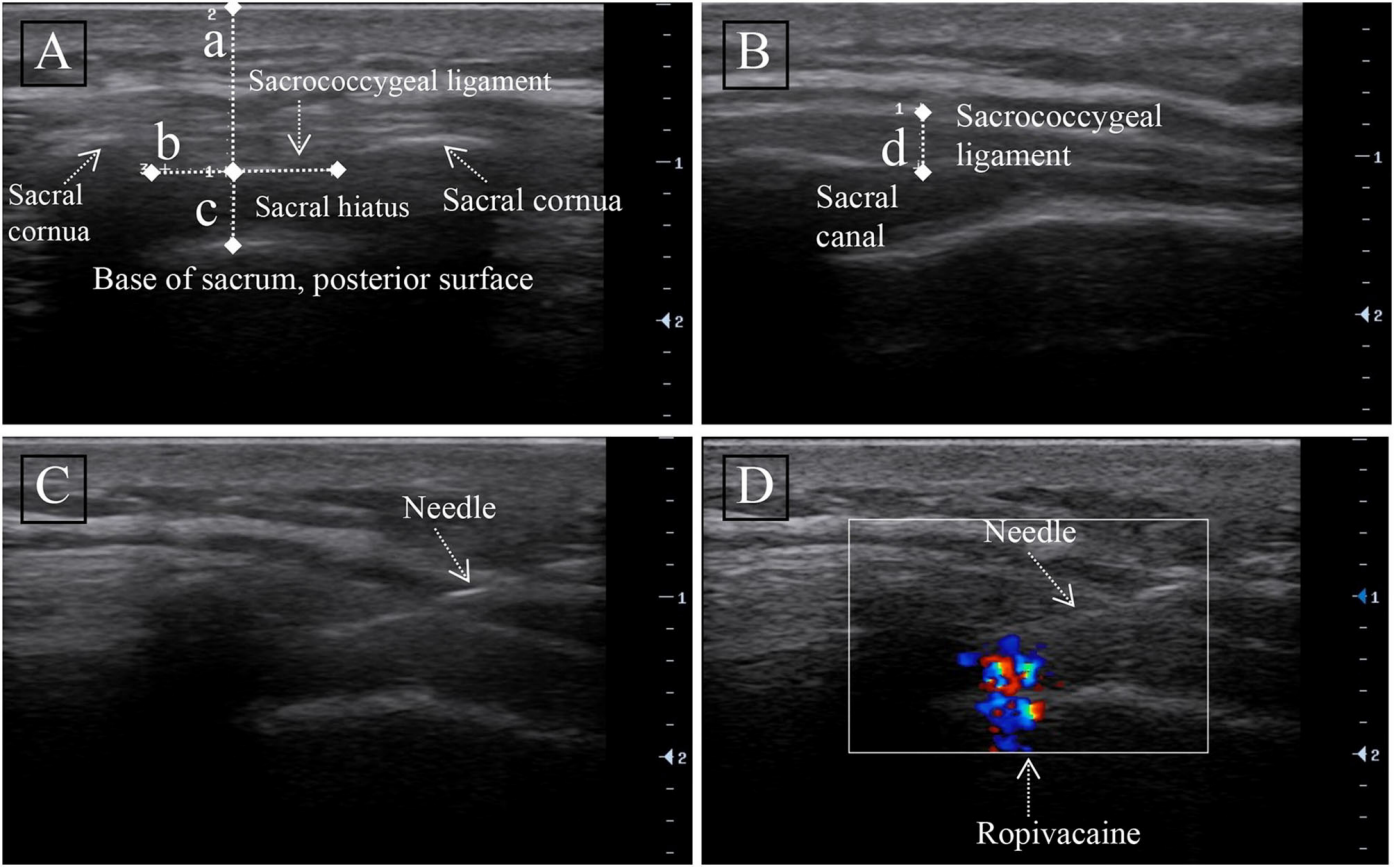
Ultrasound image of the sacral canal. **(A)** Transversal ultrasound image of the sacral canal. **(B)** Longitudinal ultrasound image of the sacral canal. Measurement of the distance from the anterior edge of the sacrococcygeal ligament (SL) to the sacrum (line c), skin to the anterior edge of sacral ligament distance (line a), sacrococcygeal ligament width (b), and thickness (line d) were done. **(C)** Longitudinal ultrasound image of the needle in the sacral canal. **(D)** Unidirectional flow on color Doppler showing the injection of ropivacaine into the sacral canal.

The sacral cornu was first touched by manipulation on the cephalic side of the gluteal fissure, and a “+” mark was made. The linear probe was then placed at the mark to search for the sacral cornu, the sacrococcygeal ligament (SL), and the sacral base, and these anatomical structures were illustrated in the transverse view (Figure 1A). The distance from the skin to the lower margin of the sacral ligament was measured (a) in the transverse view (A), the anteroposterior (AP) diameter of the sacral canal was measured at the apex of the sacral hiatus (c), and the intercornual distance between the bilateral cornu was the width of the sacral ligament (b). To obtain a longitudinal view (Figure 1B), the ultrasonic probe was rotated 90 degrees and identify the sacral ligament (SL) (Line D). The thickness of the sacral ligament was measured in the longitudinal view (B) (d).

After confirming the sacral hiatus, a 2 ml intramuscular needle (23G) was inserted out-of-plane into the sacral canal through the SL (Figure 1C). Following confirmation of needle passage through the SL into the sacral canal by loss-of-resistance to injection of <2 ml of 0.9% saline (11), color Doppler ultrasound can be used to detect successful caudal block (Figure 1D). We injected the test dose, which was 1 ml of the solution containing 5 μg of adrenaline, after negative pressure suction without blood reflux. If no evidence of intravascular injection was found after 2 min, injections of 0.5% ropivacaine (100 mg/10 ml of Naropin; AstraZeneca) were continued at a rate of 0.2 ml/S, with the volume of ropivacaine calculated from the anesthetic effect of the previous patient. Following the completion of the CEB, the patient returned to a recumbent position for further evaluation.

Following the injection of local anesthetic, a researcher (author T. Y.) tested the sensory and motor planes of the patient every 5 min for 15 min in the S1–S5 area. The researcher was unaware of the operation process and CE dosage (14). A pinprick sensation test with a blunt 16G needle was used to grade sensory block on a three-point scale: 0 indicates no block (when compared to the contralateral side); 1 indicates an incomplete block (a non-sharp sensation, touch, or pressure); and 2 indicates a complete block (unable to recognize a pinprick sensation). We considered the CEB a success if there was a complete sensory blockage in the S3–S5 area within 15 min of local anesthetic injection and if the surgeon could easily insert the anoscope. Otherwise, the CEB was deemed unsuccessful, and the anesthesia method was changed to general anesthesia.

Following confirmation of a successful CEB, 1 mg of midazolam and 0.5 μg/kg/h dexmedetomidine were given intravenously to maintain monitored anesthesia care.

After surgery, the sensory and motor blocks were assessed and recorded again in the PACU. To assess the motor block, we employed the modified Bromage scale (0 = freely moving; 1 = unable to move hip but can move knee, ankle, and toes; 2 = unable to move hip and knee but can move ankle and toes; 3 = unable to move hip, knee, and ankle but can move toes; 4 = unable to move hip, knee, ankle, and toes) (14). An independent observer (author T. Y.) followed patients postoperatively for postoperative sensory and motor recovery, postoperative urinary retention, and complications related to nerve block.

### 2.2. Biased coin design up-and-down sequential method

The biased coin design up-and-down sequential method (BCD-UDM method) was used in this study for two groups of patients (15, 16). Except for the use of 10 ml (male group) and 8 ml (female group) of 0.5% ropivacaine (17) for the first patient, the volume of nerve block of each subsequent patient is determined by the blocking effect previous patient. The ropivacaine concentration was chosen based on clinical experience and previous research. If the block is ineffective in one patient, the volume of ropivacaine in the next patient will be increased by 2 ml. There was an 11% chance that the ropivacaine volume of the next patient would be reduced by 2 ml if the patient had a better block; otherwise, there was an 89% chance that the volume would be the same as the previous patients (18).

According to previous research data, a sample size >40 was optimal for obtaining MEV90 (18). Therefore, to account for potential shedding data, this study required at least 45 positive reactions to estimate MEV90 (15). This study included 45 patients who successfully completed a block. In addition, a non-study staff member set up 44 sealed envelopes (containing the volume allocation for the successful block). We calculated MEV90 using isotonic regression and obtained 95% confidence intervals (CI) from 2,000 bootstrapping replications. We also used isotonic regression and bootstrapping to analyze CI data. To estimate the minimum volume required for sacral canal blocking in 95 and 99% of patients (15, 16), because biological and experimental variability may be unpredictable as the dose increases, the pooled-adjacent violators algorithm (PAVA), as described by Prof. Mario P. Styliano, was used to calculate the adjusted response rate (19).

Non-invasive blood pressure, electrocardiogram, and heart rate (HR) were recorded during and after CEB injection and during surgery. Hypotension was defined as a drop in systolic blood pressure of more than 30% compared to the baseline level or a drop in systolic blood pressure of <90 mmHg. If the patient had perioperative hypotension, 3 mg of ephedrine was given intravenously, along with a fluid infusion that was accelerated appropriately. Patients with bradycardia (HR < 55 beats/min) received an intravenous dose of 0.3–0.5 mg atropine.

### 2.3. Implementation and blinding

SPSS was used to generate the random allocation sequence, and each random number was sealed in a separate envelope (44 envelopes for each gender). ZP enrolled participants and assigned them to interventions based on the envelope. The 44 envelopes contained 44 randomly generated numbers ranging from 1 to 9. If the envelope was numbered 9, the next subject was assigned a volume of 2 ml less or the same volume for numbers if the envelope was numbered 1–8. MY, who was unaware of the interventions, carried out the analysis.

### 2.4. Statistics

In this study, we calculated our data using the R statistical software package version 3.2.1. We present the obtained data as the medians and interquartile ranges. We reported categorical variables as numbers (proportions) and evaluated them using Fisher's exact test or the χ^2^ test. We analyzed mean (SD) values using the unpaired Student's *t*-test or Welch *t*-test for different variances and median (interquartile) values using the Mann-Whitney *U*-test. A *P*-value of < 0.05 was considered statistically significant for all tests.

## 3. Results

**Figure 2:**
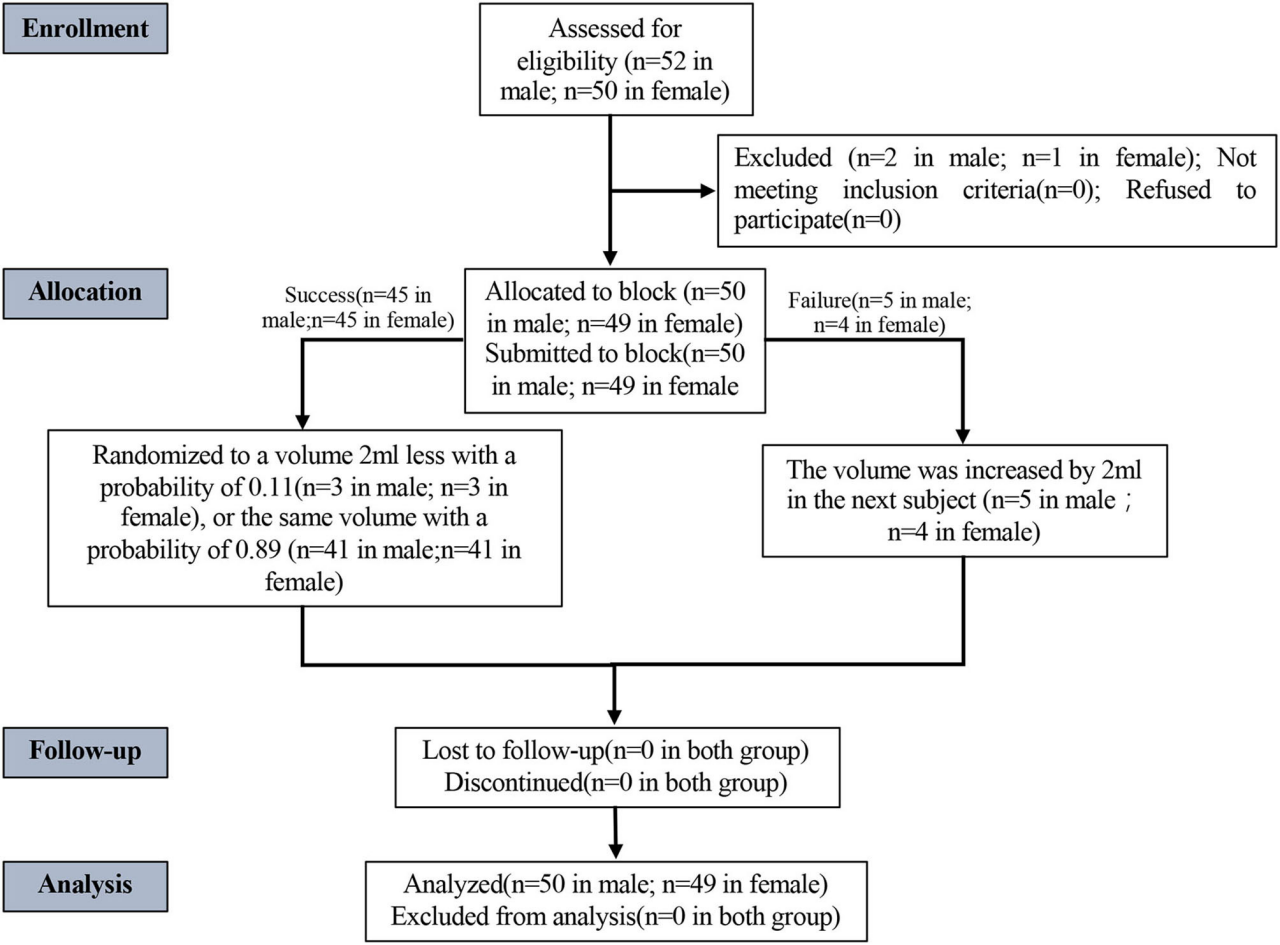
CONSORT flowchart.

The study included 102 patients (52 men and 50 women) from 1 October 2019 to 6 January 2020 (Figure 2, CONSORT flowchart). Due to sacral canal stenosis, two male patients and one female patient were excluded from the study. Finally, 50 men and 49 women (a total of 99 patients) completed the CEB. As they did not meet the criteria for a successful sacral block, five male patients and four female patients were deemed failures. The anal sphincter was flaccid and perfect during the specific performance, but there was pain during the incision or operation. As shown in Figure 2, general anesthesia was used in all nine patients who failed the CEB.

Table 1 shows the demographic characteristics of patients. Weight (*p* < 0.001) and height (*p* < 0.001) were statistically different between the male and female groups. However, there was no significant difference between the two groups in terms of other characteristics such as age, BMI, ASA, and type of surgery.

**Table 1:** Characteristics of patients.

	**Male (*n* = 50)**	**Female (*n* = 49)**	***P***
**Age (y)**	**41 (28.5–54.25)**	**38 (31.5–47)**	**0.828**
Weight (kg)	70 (62.5–80)	54(49.5–58)	0.00
Height (cm)	170 (166.75–175.25)	158(154.5–160.5)	0.00
BMI	24.48 ± 3.66	21.84 ± 3.08	0.284
ASA			0.570
I	35	36	–
II	14	13	–
III	1	0	–
**Ultrasound measurement (mm)**			
Skin to sacral canal	14.22 ± 4.41	13.34 ± 4.02	0.663
Sacral fissure depth	5.12 ± 1.35	3.84 ± 1.08	0.058
Medial spacing of sacral angle	7.10 (5.90,8.50)	9.10 (7.40,10.15)	0.000
SL thickness	4.30 ± 0.98	3.43 ± 0.91	0.556
Types of surgery			0.105
Hemorrhoids	25	32	–
Perianal abscess	7	8	–
Anal fistula	17	8	–
Anal polyp	1	0	–
Rectal polyp	0	1	–

Values are mean ± SD, median (IQR), or number (proportion) where appropriate. SL, sacrococcygeal ligament.

As demonstrated by ultrasound measurement (Figure 1), the sacral ligaments were wider in female patients than in male patients (*p* < 0.001), but their AP diameter at the apex of the sacral canal was shorter (*p* = 0.058).

**Figure 3:**
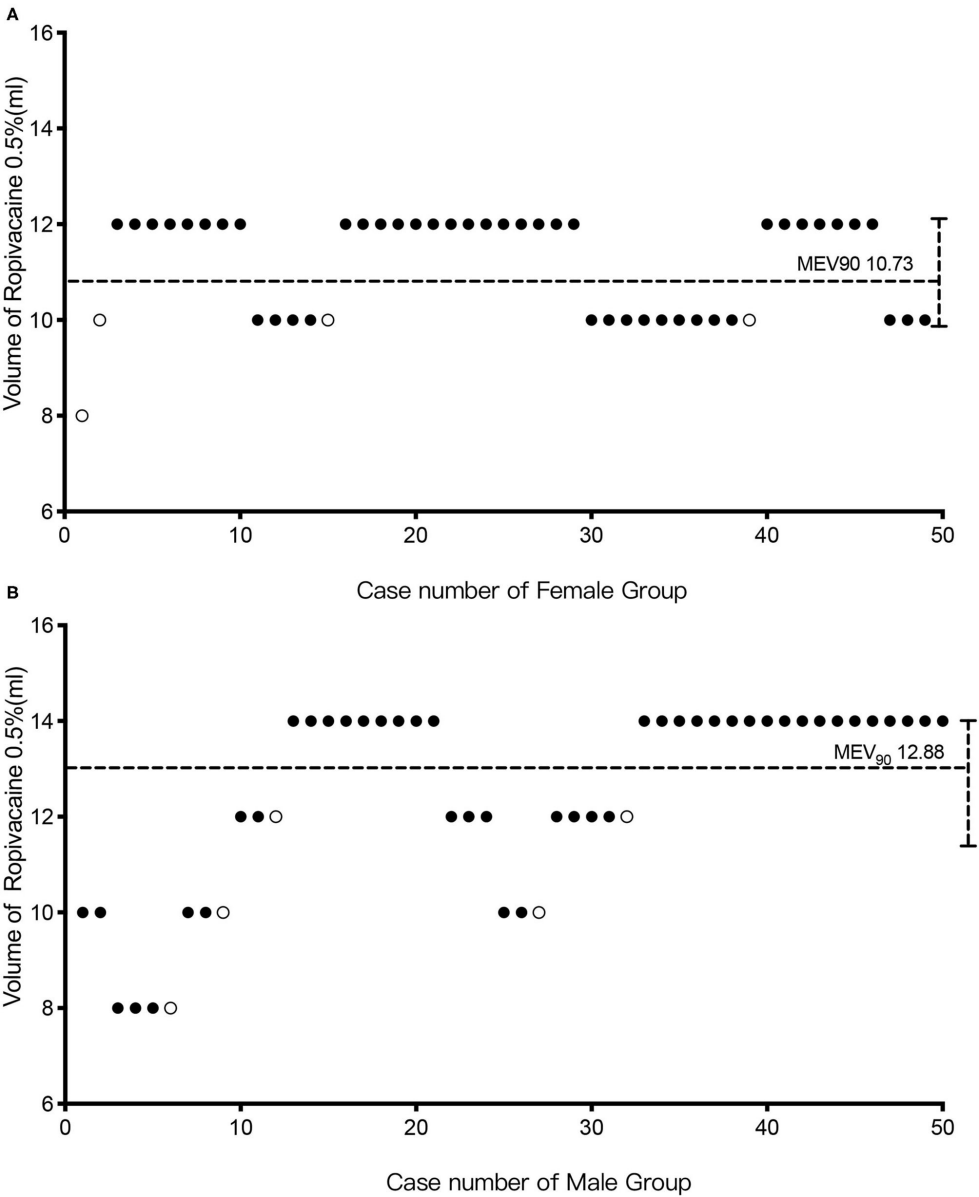
The biased coin design up-and-down sequence. Graph of successful (solid circle) and failed (hollow circle) caudal epidural blocks with different ropivacaine volumes in the female **(A)** and male groups **(B)**. The horizontal line is the calculated minimum effective volume of 0.5% ropivacaine providing successful caudal block in 90% of patients (MEV90).

Figure 3 depicts the biased coin design up-and-down sequence method. Men had a MEV90 of 12.88 ml (95% CI: 10.8–14 ml), and women had a MEV90 of 10.73 ml (95% CI: 9.67–12 ml). According to further analysis, the MEV95 was 13.44 ml (95% CI: 11.6–14 ml) for men and 11.37 ml (95% CI: 10.65–12 ml) for women, while the MEV99 was 13.88 ml (95% CI: 12.97–14 ml) for men and 11.87 ml (95% CI: 11.72–12 ml) for women.

Table 2 shows the observed response rate for each ropivacaine volume group. The response rates adjusted by the pooled-adjacent violator algorithm were also consistent with the monotonically changing response rates of the isotonic regression method.

**Table 2:** Observed and pooled-adjacent violators algorithm-adjusted response rates.

**Group**	**Assigned volume**	**Successful blocks**	**Trails**	**Observed response rate**	**PAVA-adjusted response rate**
Male	8	3	4	0.75	0.75
	10	6	8	0.75	0.75
	12	9	11	0.82	0.82
	14	27	27	1	1
Female	8	0	1	0	0
	10	16	19	0.84	0.84
	12	29	29	1	1

PAVA, pooled-adjacent violators algorithm.

Table 3 displays the general characteristics and complication data of CEB success. There was no statistical difference between the two groups in terms of anesthesia onset time, surgery duration, or postoperative pain-free duration. While providing effective analgesia, the anal sphincter was relaxed, and no motor block of both lower limbs was reported in either group. During follow-up, three patients (6.67%) in the male group were found to have low back pain, but all of them were relieved without any special treatment. There were 6 (13.33%) male patients and 6 (13.33%) female patients who experienced urinary retention after the operation and required urethral catheterization. Other serious complications did not occur in either group.

**Table 3:** Caudal epidural block characteristics and block complication.

	**Male (*n* = 45)**	**Female (*n* = 45)**	***P***
Anesthesia Onset time (min)	10.02 (8–11.5)	9.36 (6.5–11)	0.09
Operation time (min)	33.27 (25–40)	34.78 (25–40)	0.741
Postoperative pain onset time (h)	7.09 (5–8.5)	7.24 (6–9)	0.385
Urinary retention	6 (13.33%)	6 (13.33%)	1
Back pain	3 (6.67%)	0	0.242

Values are mean ± SD, median (IQR), or number (proportion) where appropriate.

**Figure 4:**
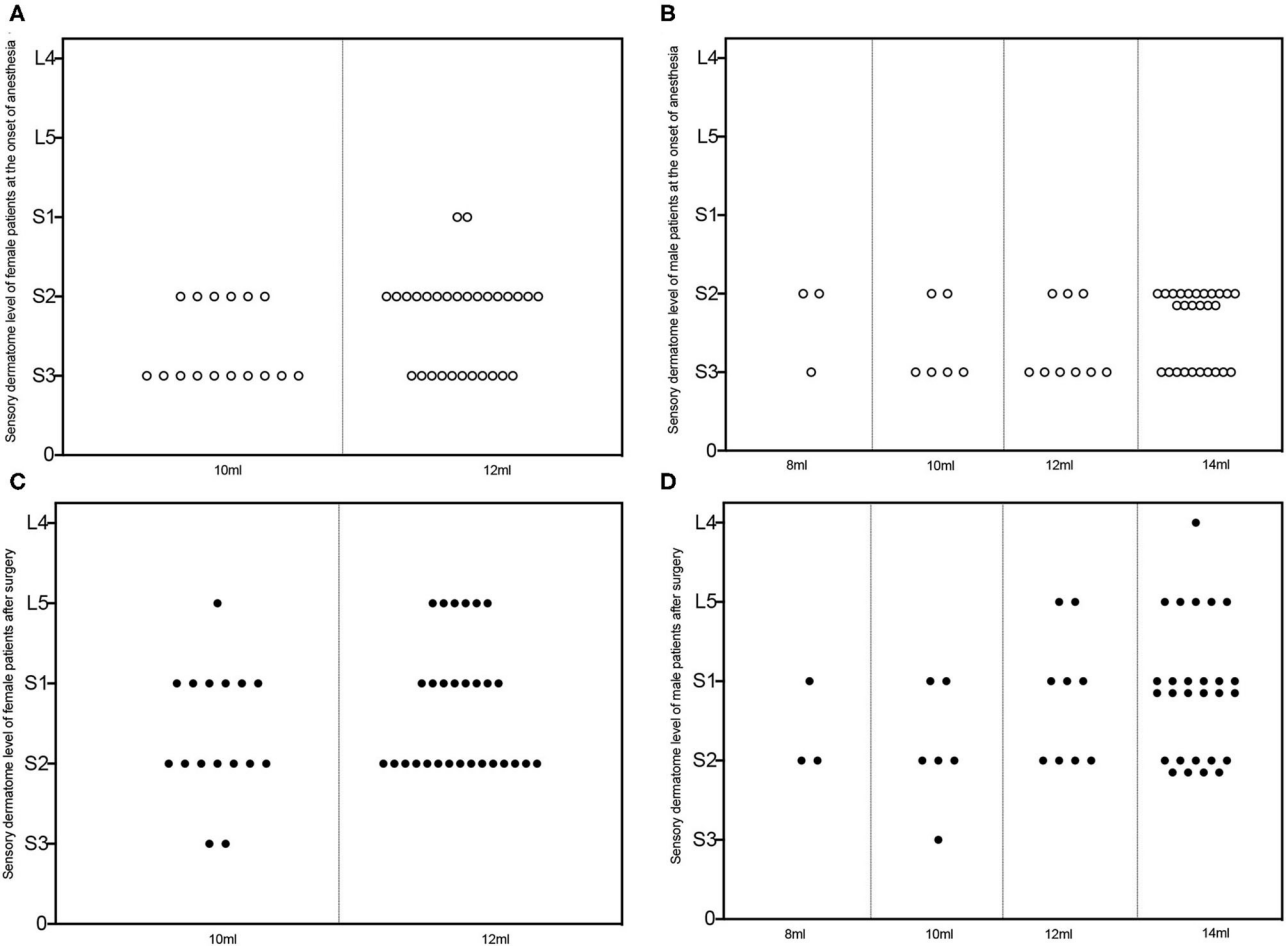
Sensory dermatome level of patients subjected to successful caudal block. Sensory dermatome levels of female **(A, C)** and male **(B, D)** patients administrated with different volumes of ropivacaine at the onset of the caudal block (hollow circle in **A, B**) and the end of surgery (solid circle in **C, D**). L, lumbar segment; S, sacral segment.

Figure 4 depicts the level of perianal sensory and motor block at the beginning and end of the surgery. The sensory and motor block levels were around S2 and S3 at the start of the surgery, and they increased to higher block levels (S2–L4) after surgery.

## 4. Discussion

The MEV90 of 0.5% ropivacaine for US-CEB for anorectal surgery was 12.88 ml for male patients and 10.73 ml for female patients, according to the BCD-UDM results. The MEV90 and MEV99 of 0.5% ropivacaine for US-CEB for anorectal surgery in adults were evaluated for the first time in this study. The findings of this study can improve patient satisfaction and comfort by increasing the success rate of the sacral block to 99% when applied to anorectal surgery, reducing related complications, allowing patients to recover to a normal state more quickly after surgery, providing a short-term analgesic effect after surgery, and, most importantly, providing a reference for clinical anesthesia (1).

In comparison to bupivacaine, ropivacaine is a long-acting amide local anesthetic that separates sensory and motor effects and is less toxic to the heart. It is widely used for CEB in both children and adults (19). With reference to the minimum effective concentration that has been used for a CEB for anorectal surgery, ropivacaine (0.1–0.5%) was widely used to maintain the caudal block (14, 20–22). To avoid CEB failure due to insufficient drug concentration rather than low volume, 0.5% ropivacaine was used to cover the entire sacral nerves in this study.

In anorectal surgery, local anesthesia infiltration, spinal anesthesia, and CEB were commonly used. Caudal block demonstrated minimal blockade, a lower degree of hypotension, less headache, better postoperative pain control without delaying discharge, and higher patient satisfaction when compared to other anesthesia methods (14, 23). Despite these benefits, clinical anesthesiologists rarely use it for anorectal surgery because even experienced anesthesiologists have only a 75% success rate with CEB (24). The traditional blinding procedure is technically difficult for adult patients. In recent years, ultrasound has gradually been applied to CEB as an important tool, and the success rate of CEB has been significantly improved. Klocke et al. first described the US-guided CEB in 2004 (21). According to Chen et al., the accuracy rate of ultrasound-guided puncture needle placement into the caudal epidural space was up to 100% (13). In comparison to contrast dye fluorescence, ultrasound is simple to use, free of radiation pollution, and can be used in almost any clinical setting (25). Most importantly, under the guidance of ultrasound, the puncture needle can be observed in real time, entering the caudal epidural space under the guidance of ultrasound, ensuring that the puncture needle enters the target position accurately (13). However, it has been reported that blocking the sacral canal under real-time ultrasound guidance is difficult if the anterior-posterior diameter (AP) of the sacral cavity at the apex of the sacral hiatus is <1.6 mm (13). We found one female and two male patients with an AP diameter <1.6 mm at the apex of the sacral hiatus who were excluded during pre-anesthesia ultrasound evaluation (4, 25). The AP diameter of the sacral canal at the apex of the sacral hiatus was 5.12 ± 1.35 (2.8–7.9) mm in male patients and 3.84 ± 1.08 (2.4–7.5) mm in female patients, according to the current study. Chen et al. found a similar value of 5.3 ± 2.1 mm after ultrasonography in 47 Taiwanese subjects.

Dixon's method, which was first used to investigate the anesthetic concentration of inhalation required to prevent the movement of the surgical incision in 50% of patients (ED50), also known as the minimum alveolar concentration, remains the mainstay method in most studies to investigate MEV50 and MEC50. The ED95 of inhaled anesthetic could be approximated from ED50 because of the steep relation of the inhaled anesthetics' concentration-response. Nonetheless, higher percentile data (such as MEV90, MEV95, MEV99, and so on) are more clinically meaningful for anesthesiologists. In contrast, the Dixon Wood sequential method extrapolates 50% of the lowest effective volume to 90% of the minimum effective volume with large errors and limited clinical value (15, 16, 26). Sequential regression is a statistical method commonly used in drug clinical trials to calculate MEV90. To further analyze data, isotonic regression and bootstrap CI can be used to estimate the minimum effective volume required for the successful blocking of 95 and 99% of patients (MEC95 and MEC99). Thus, in the current study, BCD-UDM was used for the first time to determine the 90% minimum effective volume of 0.5% ropivacaine for ultrasound-guided CEB in anorectal surgery. After that, it was extrapolated to a 99% minimum effective volume, providing an anesthesiologist with a more accurate reference.

Siddiqui et al. (14) reported that a wide range of caudal injection volumes (10–64 ml) can be used and that the volume required to reach the L5 segment is 10 ml and the volume required to reach the L4 segment is 15 ml. The diffusion of epidural injection volume was measured using epidurograms and radioactive tracers in this study (27, 28). Anne Blanchais reported that they injected a total of 20 ml of contrast medium, with 100% of the patients diffused to S1, 89% to L5, 48% to L4, and 19% to L3. The anesthesia level of the neuraxial spread is not synonymous with the physical spread of the solution measured with a contrast agent in the epidural space. The level attained by the solution is not always the plane of nerve block, and the effects of certain factors on the spread of solutions may differ from clinical experience. The volume of the sacral canal was greater in men than in women, according to a study by Asghar et al. (9), which used measurements of the cadaveric sacral lumen to conclude (9). The studies conclusions in which the capacity of the sacral lumen was determined by measuring the dry bone volume. However, the ducts are filled with the dural sac and its contents during life. The volume required for clinical anesthesia differs from the volume measured (29). This fact prompted researchers to investigate the MEV of ropivacaine, which played a role in the CEB for anorectal surgery while taking gender into account. This fact was also used to justify recommending a lower initial volume of women in this study (30, 31). The current study is the first to determine the MEV90 of 0.5% ropivacaine for the CEB for anorectal surgery using the extent of the neural blockade as the primary evaluation index.

The canal was filled with the dural sac and its contents, nerves, blood vessels, fat, and connective tissues (25), all of which influenced the degree of CEB. Some external factors also have an impact on the success of CEB. The apex of the sacral hiatus was searched by ultrasound in the current study, and the puncture was performed at an angle of 60–90° using an out-of-plane ultrasound-guided approach (28). The needle was observed under real-time ultrasound guidance penetrating through the sacral ligaments to reach the sacral canal, ensuring sacral puncture success (32).

The patient in the current study underwent anorectal surgery in the lithotomy position. The local anesthetic drugs in the sacral canal spread further cephalad due to the gravity of the body position (10, 33), which can shorten the onset time of CEB. Secondary horizontal redistribution and longitudinal cranial spread were discovered in a study and could be caused by rebound cerebrospinal fluid displacement and epidural pressure changes, resulting in differences in the initial and final levels of a block (27). The assessment of anesthetic efficacy was tested with the patient in a lithotomy position in the current study, and we expect the CEB to be fully effective when the surgeon incises the skin (28).

The sacral block should be used in anorectal surgery, and no other anesthetic drugs or modalities should be used intraoperatively. In this study, we discovered that the CEB could provide postoperative analgesia for ~7 h during anorectal surgery that other anesthetic modalities cannot and that the male and female groups are similar in this regard. If adjuvants are added to local anesthetic medications, the duration of postoperative analgesia with sacral blockade may be extended. Patients can finish eating, urinating, and receiving pain relief during this time. In this study, the volume of injectable local anesthetic used allowed the plane of anesthesia to reach only L5–S1, which can meet the need for anorectal surgery while also lowering the incidence of postoperative complications such as dyskinesia of both lower limbs and urinary retention. Lower limb motor disability was not present in either group. The most common complaint of patients with CEB was urinary retention; 13.33% of the patients in this study had urinary retention, and there was no difference between men and women. According to Yokoyama et al., 13.7% of patients developed urinary retention after hemorrhoidectomy under spinal anesthesia (28). Back pain occurred in three cases despite no special treatment.

Therefore, US-guided CEB was a relatively safe and effective anesthesia option for adults undergoing anorectal surgery. The pooled-adjacent violator algorithm-adjusted analysis in Table 1 revealed that CEB in anorectal surgery was safe and effective with 14 ml of 0.5% ropivacaine for male patients and 12 ml of 0.5% ropivacaine for female patients.

This study had some limitations. First, all CEBs were performed by a single anesthesiologist, which may limit the generalizability of the findings of the study. Second, the definition of a successful CEB in this study lacked objective and quantitative indicators and relied on surgeon judgment. The relaxation of perianal muscles may differ depending on the opinion of the surgeon. Anal sphincter tension detection may be an objective strategy for assessing the degree of anal sphincter relaxation. Furthermore, the BMI of all patients in this study is <30 kg/m^2^, which should be applicable to patients with a high BMI. As high BMI makes CEB more difficult but the sacral cavity is a bony structure whose capacity does not increase with weight, existing issues must be researched further (17).

## 5. Conclusion

Finally, the findings of this study have the potential to increase the success rate of CEB for anorectal surgery to 99% with operability and general applicability while decreasing the incidence of anesthesia-related complications. CEB can meet the needs of patients for rapid postoperative rehabilitation, improve patient satisfaction, and lay a solid foundation for postoperative analgesia.

## Data availability statement

The raw data supporting the conclusions of this article will be made available by the authors, without undue reservation.

## Ethics statement

The studies involving human participants were reviewed and approved by Clinical Trial Ethics Committee of Chengdu Shangjin Nanfu Hospital. The patients/participants provided their written informed consent to participate in this study.

## Author contributions

Material preparation, data collection, and analysis were performed by PZ, XH, JL, and MY. Conceptualization: XL and PZ. Methodology: XL. Formal analysis and investigation: PZ and MY. Writing original draft: XL, TY, and PZ. Revising the draft: PZ and HC. Writing—review and editing and funding acquisition: XL and RW. Resources and supervision: RW. All authors contributed to the conception and design of the study and read and approved the final manuscript.

## References

[1] Sammour T Barazanchi AW Hill AG PROSPECT Group. Evidence-based management of pain after excisional haemorrhoidectomy surgery: a PROSPECT review update. World J Surg. (2017) 41:603–14. 10.1007/s00268-016-3737-1 27766395

[2] Nikooseresht M Hashemi M Mohajerani SA Shahandeh F Agah M. Ultrasound as a screening tool for performing caudal epidural injections. Iran J Radiol. (2014) 11:e13262. 10.5812/iranjradiol.13262 25035698 PMC4090639

[3] Kao SC Lin CS. Caudal epidural block: an updated review of anatomy and techniques. Biomed Res Int. (2017) 2017:9217145. 10.1155/2017/9217145 28337460 PMC5346404

[4] Kim DH Park JH Lee SC. Ultrasonographic evaluation of anatomic variations in the sacral hiatus: implications for caudal epidural injections. Spine. (2016) 41:E759–63. 10.1097/BRS.0000000000001448 27340767

[5] Simpson D Curran MP Oldfield V Keating GM. Ropivacaine: a review of its use in regional anaesthesia and acute pain management. Drugs. (2005) 65:2675–717. 10.2165/00003495-200565180-00013 16392884

[6] Dene Simpson MPC Vicki O Gillian MK. Ropivacaine a review of its use in regional anaesthesia and acute pain management. Drugs. (2005) 65:2675–717. 16392884 10.2165/00003495-200565180-00013

[7] Gan S Song L Chen W Feng Z Li Y Zhang J. Strength and sensation after epidural ropivacaine in men and women. Anaesthesia. (2015) 70:1060–5. 10.1111/anae.13085 25919788

[8] Wiegele M Marhofer P Lonnqvist PA. Caudal epidural blocks in paediatric patients: a review and practical considerations. Br J Anaesth. (2019) 122:509–17. 10.1016/j.bja.2018.11.030 30857607 PMC6435837

[9] Asghar A Naaz S. The volume of the caudal space and sacral canal in human sacrum. J Clin Diagn Res. (2013) 7:2659–60. 10.7860/JCDR/2013/6287.3724 24551603 PMC3919357

[10] Park GY Kwon DR Cho HK. Anatomic differences in the sacral hiatus during caudal epidural injection using ultrasound guidance. J Ultrasound Med. (2015) 34:2143–8. 10.7863/ultra.14.12032 26491092

[11] Aggarwal A Aggarwal A Harjeet Sahni D. Morphometry of sacral hiatus and its clinical relevance in caudal epidural block. Surg Radiol Anat. (2009) 31:793–800. 10.1007/s00276-009-0529-4 19578805

[12] Kavakli AS Kavrut Ozturk N Arslan U. Minimum effective volume of bupivacaine 05% for ultrasound-guided retroclavicular approach to infraclavicular brachial plexus block. Braz J Anesthesiol. (2019) 69:253–8. 10.1016/j.bjane.2018.12.011 31030903 PMC9391849

[13] Chen CP Wong AM Hsu CC Tsai WC Chang CN Lin SC. Ultrasound as a screening tool for proceeding with caudal epidural injections. Arch Phys Med Rehabil. (2010) 91:358–63. 10.1016/j.apmr.2009.11.019 20298824

[14] Siddiqui ZI Denman WT Schumann R Hackford A Cepeda MS Carr DB. Local anesthetic infiltration versus caudal epidural block for anorectal surgery: a randomized controlled trial. J Clin Anesth. (2007) 19:269–73. 10.1016/j.jclinane.2006.12.004 17572321

[15] Stylianou M Flournoy N. Dose finding using the biased coin up-and-down design and isotonic regression. Biometrics. (2002) 58:171–7. 10.1111/j.0006-341X.2002.00171.x 11890313

[16] Stylianou M Proschan M Flournoy N. Estimating the probability of toxicity at the target dose following an up-and-down design. Stat Med. (2003) 22:535–43. 10.1002/sim.1351 12590412

[17] Li X Li J Zhang P Deng H Yang M He H. The minimum effective concentration (MEC90) of ropivacaine for ultrasound-guided caudal block in anorectal surgery. A dose finding study. PLoS ONE. (2021) 16:e0257283. 10.1371/journal.pone.0257283 34534232 PMC8448308

[18] Fang G Wan L Mei W Yu HH Luo AL. The minimum effective concentration (MEC90) of ropivacaine for ultrasound-guided supraclavicular brachial plexus block. Anaesthesia. (2016) 71:700–5. 10.1111/anae.13445 26945818

[19] Pace NL Stylianou MP. Advances in and limitations of up-and-down methodology: a précis of clinical use, study design, and dose estimation in anesthesia research. Anesthesiology. (2007) 107:144–52. 10.1097/01.anes.0000267514.42592.2a 17585226

[20] Higuchi H Adachi Y Kazama T. Factors affecting the spread and duration of epidural anesthesia with ropivacaine. Anesthesiology. (2004) 101:451–60. 10.1097/00000542-200408000-00027 15277929

[21] Li Y Zhu S Bao F Xu J Yan X Jin X. The effects of age on the median effective concentration of ropivacaine for motor blockade after epidural anesthesia with ropivacaine. Anesth Analg. (2006) 102:1847–50. 10.1213/01.ane.0000215999.60513.da 16717335

[22] Higuchi H Hirata J Adachi Y Kazama T. Influence of lumbosacral cerebrospinal fluid density, velocity, and volume on extent and duration of plain bupivacaine spinal anesthesia. Anesthesiology. (2004) 100:106–14. 10.1097/00000542-200401000-00019 14695731

[23] Vadhanan P Rajendran I Rajasekar P. Ultrasound-guided caudal epidural anesthesia in adults for anorectal procedures. Anesth Essays Res. (2020) 14:239–42. 10.4103/aer.AER_60_20 33487822 PMC7819400

[24] Sekiguchi M Yabuki S Satoh K Kikuchi S. An anatomic study of the sacral hiatus: a basis for successful caudal epidural block. Clin J Pain. (2004) 20:51–4. 10.1097/00002508-200401000-00010 14668657

[25] Hazra AK Bhattacharya D Mukherjee S Ghosh S Mitra M Mandal M. Ultrasound versus fluoroscopy-guided caudal epidural steroid injection for the treatment of chronic low back pain with radiculopathy: a randomised, controlled clinical trial. Indian J Anaesth. (2016) 60:388–92. 10.4103/0019-5049.183391 27330199 PMC4910477

[26] Li Y Zhou Y Chen H Feng Z. The effect of sex on the minimum local analgesic concentration of ropivacaine for caudal anesthesia in anorectal surgery. Anesth Analg. (2010) 110:1490–3. 10.1213/ANE.0b013e3181d6bade 20304981

[27] Lundblad M Eksborg S Lonnqvist PA. Secondary spread of caudal block as assessed by ultrasonography. Br J Anaesth. (2012) 108:675–81. 10.1093/bja/aer513 22315327

[28] Yokoyama M Hanazaki M Fujii H Mizobuchi S Nakatsuka H Takahashi T. Correlation between the distribution of contrast medium and the extent of blockade during epidural anesthesia. Anesthesiology. (2004) 100:1504–10. 10.1097/00000542-200406000-00024 15166571

[29] Park WY Massengale M Macnamara TE. Age, height, and speed of injection as factors determining caudal anesthetic level, and occurrence of severe hypertension. Anesthesiology. (1979) 51:81–4. 10.1097/00000542-197907000-00019 453598

[30] Ciccone GK Holdcroft A. Drugs and sex differences: a review of drugs relating to anaesthesia. Br J Anaesth. (1999) 82:255–65. 10.1093/bja/82.2.255 10365004

[31] Nofal WH Abdelaal WA Elfawal SM. Minimum effective volume of bupivacaine in spinal anesthesia for elective cesarean section. Does it differ with height? A non-randomized parallel study. Egypt J Anaesth. (2019) 33:67–72. 10.1016/j.egja.2016.10.008

[32] Senoglu N Senoglu M Oksuz H Gumusalan Y Yuksel KZ Zencirci B. Landmarks of the sacral hiatus for caudal epidural block: an anatomical study. Br J Anaesth. (2005) 95:692–5. 10.1093/bja/aei236 16155035

[33] Crighton IM Barry BP Hobbs GJ. A study of the anatomy of the caudal space using magnetic resonance imaging. Br J Anaesth. (1997) 78:391–5. 10.1093/bja/78.4.391 9135359

